# Supplementary material for: Analysis of cerebral Interleukin-6 and tumor necrosis factor alpha patterns following different ventilation strategies during cardiac arrest in pigs
Source: PeerJ. 2023 Sep 29;11:e16062. doi: 10.7717/peerj.16062 (PMC10544304; doi:10.7717/peerj.16062)
Supplement: Supplemental Information 1 [file peerj-11-16062-s001.docx]

|  | | N | Mean | Standard deviation |
| --- | --- | --- | --- | --- |
|  |  |  |  |  |
| p_a_CO_2_ | IPPV | 87 | 45,7345 | 11,89617 |
|  | SV 20mbar | 87 | 48,9437 | 19,38394 |
|  | CCSV 40mbar | 62 | 51,2887 | 23,51004 |
|  | Overall | 236 | 48,3767 | 18,37681 |
| p_a_O_2_ | IPPV | 87 | 155,7092 | 48,90125 |
|  | SV 20 | 87 | 143,6092 | 54,76911 |
|  | CCSV 40 | 62 | 186,9548 | 129,95608 |
|  | Overall | 236 | 159,4572 | 81,57443 |


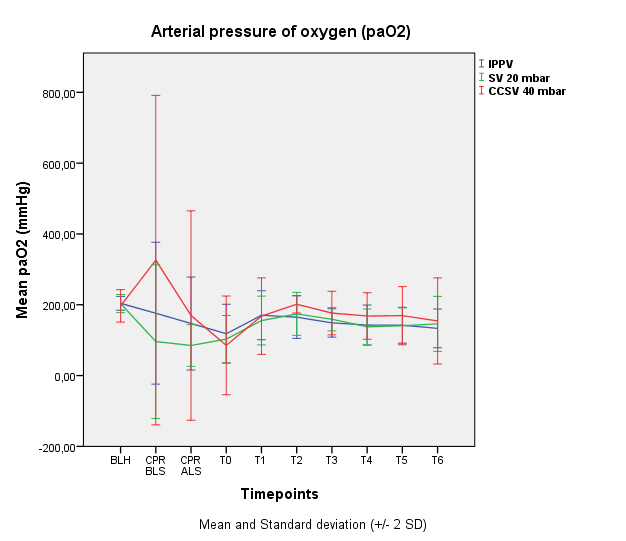


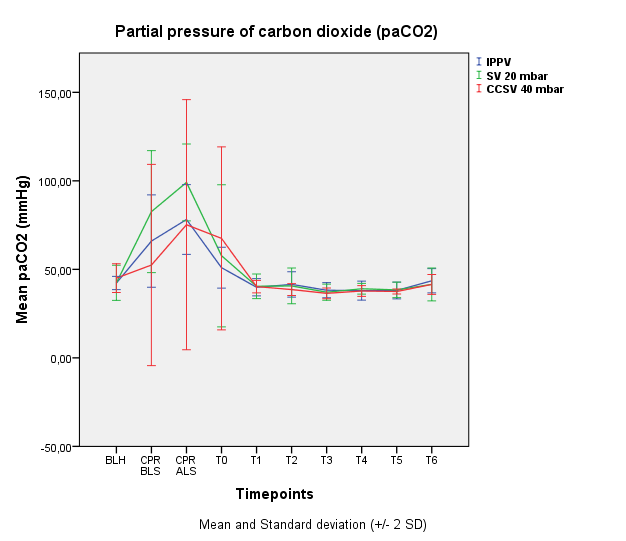


The BGAs showed a trend towards lower PaO_2_ and higher PaCO_2_ in the SV 20 group during basic life support, especially when compared to the CCSV 40 group. However, variance was very high, which is why no statistical significance was reached.
